# Annotation of biological samples data to standard ontologies with support from large language models

**DOI:** 10.1016/j.csbj.2025.05.020

**Published:** 2025-05-26

**Authors:** Andrea Riquelme-García, Juan Mulero-Hernández, Jesualdo Tomás Fernández-Breis

**Affiliations:** Departamento de Informática y Sistemas, Universidad de Murcia, CEIR Campus Mare Nostrum, IMIB-Pascual Parrilla, Murcia, 30100, Spain

**Keywords:** Bioinformatics, Generative AI, Large language models, Data interoperability, Biological samples

## Abstract

The semantic integration of biological data is hindered by the vast heterogeneity of data sources and their limited semantic formalization. A crucial step in this process is mapping data elements to ontological concepts, which typically involves substantial manual effort. Large Language Models (LLMs) have demonstrated potential in automating complex language-related tasks and may offer a solution to streamline biological data annotation. This study investigates the utility of LLMs—specifically various base and fine-tuned GPT models—for the automatic assignment of ontological identifiers to biological sample labels. We evaluated model performance in annotating labels to four widely used ontologies: the Cell Line Ontology (CLO), Cell Ontology (CL), Uber-anatomy Ontology (UBERON), and BRENDA Tissue Ontology (BTO). Our dataset was compiled from publicly available, high-quality databases containing biologically relevant sequence information, which suffers from inconsistent annotation practices, complicating integrative analyses. Model outputs were compared against annotations generated by text2term, a state-of-the-art annotation tool. The fine-tuned GPT model outperformed both the base models and text2term in annotating cell lines and cell types, particularly for the CL and UBERON ontologies, achieving a precision of 47–64% and a recall of 88–97%. In contrast, base models exhibited significantly lower performance. These results suggest that fine-tuned LLMs can accelerate and improve the accuracy of biological data annotation. Nonetheless, our evaluation highlights persistent challenges, including variable precision across ontology categories and the continued need for expert curation to ensure annotation validity.

## Highlights

• The best fine-tuned model (GPT-4o-mini) achieved precision ranging from 14% to 59%.
• The precision of the model varies depending on the ontology.
• The use of the descriptions of the labels as context did not yield better results.
• The best results were obtained for cell lines and cell types.
• Better performance than tools such as text2term for cell lines and cell types.

## Graphical abstract

**Figure:**
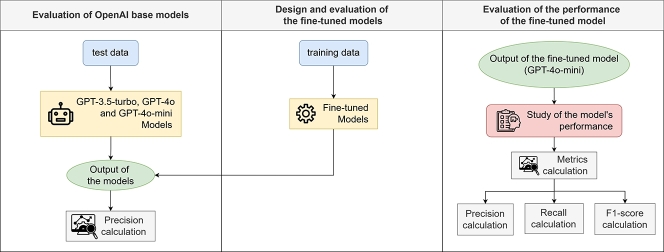

## 1 Introduction

The semantic integration of biological data is challenged by the heterogeneity of data sources, lack of standardization, and evolving ontologies. This issue is especially pronounced in biomedicine, where data is often compartmentalized by specialty and presented in diverse, non-interoperable formats [1], [2], [3], [4], [5]. Such fragmentation hinders data discovery, analysis, and reuse, even for simple research questions. Enhancing data interoperability through networked connections between resources could greatly benefit fields like precision medicine [6], [7].

As the volume of publicly available data grows, there is a growing demand for systems that can efficiently manage and utilize these resources [8]. Adhering to the FAIR (Findable, Accessible, Interoperable, Reusable) principles [9] is key, and annotating data with ontology entities supports this goal. These principles emphasize machine-readability to enable automated data processing, which is increasingly necessary given the complexity and scale of modern scientific data [9], [10].

Knowledge Graphs (KGs) are representations of heterogeneous data based on the principles of linked open data and the semantic web, providing an infrastructure composed of machine-comprehensible content [11]. This has allowed them to be used for a long time in life sciences, due to their ability to represent the complex interconnected systems studied in this field effectively [12]. Annotating an entity to an ontology or KG is the process of linking or tagging that entity (for example, a biological concept) with one or more terms or classes from a formal ontology. The annotation of biological terms to an ontology or KG, reduces data redundancy and improves interoperability between them, facilitating and providing additional information in various research fields, as biomedical information inherently contains many relationships that can be exploited to gain new knowledge.

An example of biological KG based is BioGateway [7], [13], designed to facilitate the analysis and integration of large volumes of biological data, providing access to these integrated data using semantic web technologies. BioGateway includes data from 38 publicly accessible resources about several biological domains of interest such as genes, proteins, gene ontology annotations (biological processes, cellular components, and molecular functions), phenotypes, cis-regulatory modules, topologically associated domains, multiple taxa, and different types of relationships between domains, including protein-protein interactions or regulatory relations at different levels, among others.

Despite there are standards in biology, such as the use of Gene Symbols for genes, this is not common in many biological subdomains. Typically, multiple types of representation are employed in different resources, where the use of name labels instead of appropriate identifiers is a common situation [3]. This is the case of biological samples, which are named and annotated in sources without a consensus. Biological samples have an essential role in biological processes since complex organisms exhibit functional specialization.

Among the methods proposed in the state of the art of the annotation of labels to ontology terms, we highlight text2term [14]. The text2term tool is designed to map free-text descriptions of biomedical terms to controlled ontology terms, addressing the challenge of inconsistent metadata in biomedical data repositories. It supports multiple mapping methods, including the edit distance and the Term Frequency-Inverse Document Frequency (TF-IDF) metrics, and interfaces with BioPortal and Zooma Annotators [14].

Despite their efficacy in specific domains, tools such as text2term depend excessively on surface-level lexical similarity, manifest discernible limitations. While these tools tend to perform well when dealing with terms that possess rich linguistic descriptions, their accuracy is often diminished in contexts where terminology is sparse, opaque, or heavily abbreviated. Examples of such situations are labels following naming conventions based on legacy codes, alphanumeric identifiers, or community-specific abbreviations. The inability of these tools to contextualize or infer semantics beyond direct string comparisons prevents them from capturing the nuanced associations necessary for accurate annotation in those contexts. Therefore, the development of annotation methods overcoming such limitations is needed.

We believe that the availability of methods and tools for supporting the annotation of biomedical data would be useful for different application scenarios, among which we highlight: (1) ensuring data interoperability between disparate systems and databases; (2) ensuring data standardization in scenarios in which multiple terminologies are frequently used; (3) facilitating natural language processing of medical records by associating strings describing for example diagnoses with existing Internationalized Resource Identifiers (IRIs) in resources such as SNOMED CT^1^; and (4) optimization of semantic search engines.

Large Language Models (LLMs) show significant promise for automating the annotation of natural language texts with ontology identifiers [15]. OpenAI's GPT models have led this advancement, demonstrating near-human performance in various cognitive tasks. Open-source models like Llama-3 are also emerging as strong alternatives [16], [17], [18]. LLMs are increasingly used in medicine [19] for tasks such as structuring and categorizing information, information curation [20], question answering [21], [22], supporting diagnosis [23], [24], biomedical simulation [25], interview analysis [26], and biomedical text processing tasks [15], [27]. However, their potential for annotating complex biological data remains underexplored, and further research is needed to assess their effectiveness in knowledge acquisition and concept normalization within biomedical contexts.

The utilization of LLMs for entity-to-ontology annotation confers a substantial advantage over conventional term-matching tools, owing to their capacity to capture comprehensive contextual semantics. Contrary to methodologies that predominantly depend on string similarity or predefined lexicons, LLMs possess the capability to disambiguate entities based on their utilization within a particular context. This capacity enables a more precise alignment with ontological frameworks, even in scenarios where explicit lexical matches are not available. Furthermore, LLM-based workflows seamlessly integrate with contemporary AI and NLP pipelines, facilitating scalable and modular development.

The objective of this work is to investigate the potential of LLMs for the precise, one-to-one mapping of biological sample labels to existing ontological identifiers, ensuring that each label is linked to a single, unambiguous concept from a target ontology. We evaluate the performance of both base and fine-tuned OpenAI GPT models on this task using four well-established ontologies. This challenge is critical, as biological databases often employ heterogeneous labels to describe the same underlying entities across repositories, leading to inconsistencies that hinder semantic integration. Our approach emphasizes deterministic identifier assignment, rather than probabilistic class membership, to support interoperability in scenarios where unambiguous entity resolution is essential, such as cross-database queries or meta-analyses that rely on shared ontological references.

The primary contribution of our research is the evaluation of LLMs' capacity for annotating biological samples, thereby providing a more comprehensive and unified perspective on the biological data that underlies progress in biomedical research.

## 2 Methods

### 2.1 Dataset

For the purpose of this study, a sample of the existing landscape in the domain was obtained by acquiring the labels or names of biological samples from 27 publicly available biological databases (see Supplementary Table 1). A total of 6264 labels were collected (see the “*biosamples.tsv*” file included in the GitHub repository BiosamplesLLMAnnotation^2^), and then manually classified into three different biosample types, or type of concept: cell lines, cell types, and anatomical structures (see Table 1). However, the categorization of all biological samples was not possible due to the cryptic nature of the labels and the paucity of detailed information.

**Table 1:** Number of labels by concept type in the initial dataset.

Type of concept	Number of labels
Cell Line	3080
Cell Type	2258
Anatomical structure	723
No concept	203
**Total**	**6264**

The dataset obtained was quite repetitive, containing different variations of the same sample label, although strictly duplicate labels were removed. This redundancy originates from the original data sources, reflecting the fragmented nature of current biological data repositories.

Furthermore, to establish a gold standard, each biological sample was manually annotated to concepts from four reference domain ontologies belonging to the Open Biological and Biomedical Ontology (OBO) Foundry [28]:

• *Cell Line Ontology* (CLO) [29]: Community-driven resource within the domain of biological cell lines, specifically emphasizing permanent cell lines derived from culture collections.
• *Cell Ontology* (CL) [30]: Structured, controlled vocabulary developed to categorize and define various cell types.
• *Uber-anatomy Ontology* (UBERON) [31]: Comprehensive, cross-species anatomy ontology that organizes a wide range of anatomical entities based on traditional criteria, including structure, function, and developmental lineage.
• *BRENDA Tissue Ontology* (BTO) [32]: Structured, controlled vocabulary detailing enzyme sources, encompassing terms for tissues, cell lines, cell types, and cell cultures from both unicellular and multicellular organisms.

The selection of these ontologies was predicated on their established maturity and popularity within the domain of biological ontologies. For instance, they are the ontologies that have been put forth as the optimal selection by the BioLink schema, a model for the life sciences designed to promote interoperability between biomedical KGs [33].

Finally, the ‘*biosamples.tsv*’ file also contains the human reference annotations that will be used in this work to train and evaluate the LLMs (see Table 2).

**Table 2:** Example of the data. The initial column corresponds to the label of the biological sample in the original database. The subsequent columns, from the second to the fifth, denote the identifiers corresponding to the annotation of the biological samples in the relevant ontologies. The final column designates the nature of the concept linked to the label. In this particular example, three cell lines are shown, reported as CL.

Biosample label	CLO ID	CL ID	UBERON ID	BTO ID	Type of concept
226LDM_normal_breast_luminal_cells	-	CL_0002326	UBERON_0000310	-	CL
22Rv1	CLO_0001200	CL_0002231	UBERON_0002367	BTO_0002999	CL
22rv1-arvs	CLO_0001200	CL_0002231	UBERON_0002367	BTO_0002999	CL

### 2.2 OpenAI GPT

GPT models were utilized through the OpenAI API, which operates on a pay-as-you-go pricing model. Under this model, users are charged solely for their API requests. Prior to the API's processing of a request, the input and output of the model are divided into tokens. Each model is able to process a maximum number of tokens. Next, we describe the different base models and fine-tuned models used in this work.

#### 2.2.1 The base models

The OpenAI API leverages a variety of models, each offering distinct capabilities [34] (Table 3). We have used the following GPT models:

• **GPT-3.5-turbo-0125**: It balances computational efficiency with high-quality text generation, suitable for tasks requiring quick responses and lower resource consumption. It was fine-tuned for a wide range of conversational and generative tasks with improved scalability.
• **GPT-4-turbo-2024-04-09**: It provides a more advanced and efficient version of GPT-4, offering enhanced reasoning, context retention, and accuracy.
• **GPT-4o-2024-08-06**: GPT-4o (“o” for “omni”) maintains the high intelligence level of GPT-4-turbo, but operates with significantly greater efficiency.
• **GPT-4o-mini-2024-07-18**: This model is a lightweight version requiring fewer resources, making it ideal for fast, scalable applications.

**Table 3:** Characteristics of the GPT models used.

Model	Context window	Max Output Tokens	Training Data
GPT-3.5 Turbo	16,385 tokens	4,096 tokens	Up to Sep 2021
GPT-4 Turbo	128,000 tokens	4,096 tokens	Up to Dec 2023
GPT-4o	128,000 tokens	16,384 tokens	Up to Oct 2023
GPT-4o-mini	128,000 tokens	16,384 tokens	Up to Oct 2023

In the annotation process supported by the LLM, the key point lies in the **prompt**, through which interaction with these artificial intelligence services occurs. The three base GPT models utilized in this study, namely, GPT-3.5, GPT-4, and GPT-4o, demanded a prompt that encompassed instances of the desired output format, in addition to the context within which the task was to be executed. Prefacing the textual instructions and exemplifications of the desired interaction within the prompt has been demonstrated to enhance the output of the LLM by improving the model's comprehension of the task context [35].

The prompt used in this work (see *“prompt_search_id.txt”* in our GitHub repository) is structured into five components, which we explain next:

• **Role**: The role delineates the model's expected posture for the task at hand. In this particular instance, the model is instructed to adopt the role of an expert ontology annotator.
• **Objectives**: The objectives inform the model of the task to be performed and its context. The main objective is to search for suitable identifiers for each of the labels in the four ontologies under study, knowing that the labels will be related to biological samples such as cell lines, cell types, or anatomical structures.
• **Inputs**: The inputs correspond to the external data input. In this particular instance, the sole input is the label that requires annotation.
• **Process Refinements**: This involves specifying the desired output format of the data to the model.
• **Constraints**: The constraints delineate the limitations that the model is required to consider during the presentation of the output. In this particular instance, the constraints are designed to guarantee that the model produces solely a list of identifiers, devoid of any introductory text, explanations, or conclusions.

After the prompt has been drafted, it is transmitted via the OpenAI API to the designated model, and the output is obtained. This output comprises the specific identifiers for each test data label of interest, formatted according to the established requirements.

#### 2.2.2 The fine-tuned models

Fine-tuning is a training technique that involves reusing predefined and pre-trained artificial network architectures with the idea of adjusting the pre-trained model to a specific dataset, thereby leveraging its prior learning [34], [36]. We performed a fine-tuning of the **GPT-3.5**, **GPT-4o** and **GPT-4o-mini** models. From the “*biosamples.tsv*” file that contains 6264 labels with their respective annotations in each of the ontologies, a **training data subset** was obtained for the subsequent fine-tuning process (52.5% of the data / 3288 labels), a **validation data subset** used during the model development process to adjust hyperparameters (17.5% of the data / 1096 labels), and finally, a **test data subset** for model performance evaluation (30% of the data / 1880 labels). The three data subsets were obtained by random selection.

The fine-tuning job was initiated using the training and validation data subsets with the following hyperparameters: epochs = 6; batch size = 3; and learning rate multiplier = 0.3. As mentioned above, the dataset contains concepts represented with different labels, which will also be present in the training dataset. This enables the model to discern that labels with spelling variations can denote the same real-world entity. Thus, three new fine-tuned models were created with the data of interest from the GPT-3.5, GPT-4o, and GPT-4o-mini models. In contrast to the base models, the prompt in these new models has been reduced to a single sentence. This reduction is due to the fact that the context of the task and the output format have been previously taught during the fine-tuning process.

Furthermore, an additional experiment was conducted in which the dataset utilized for fine-tuning included not only the sample labels and their identifiers but also their descriptions. The objective was to investigate whether providing additional context would enhance the model's performance on the annotation task.

### 2.3 Evaluation method

The performance of the model has been evaluated in three ways. First, the annotations suggested by the model were compared to a gold standard annotation, thus generating a series of evaluation metrics (see Section 2.3.1). Second, a second human annotated a set of labels and we compared the inter-expert agreement, which can be used to revisit the performance of the model (see Section 2.3.2). Finally, we explain how we have compared the results of the model with the ones obtained by a state-of-the-art tool that applies traditional methods such as text2term [14] (see Section 2.3.3).

#### 2.3.1 Metrics for the evaluation of the models

To assess the performance of the models, predictions are classified as true positives (TP), false positives (FP), false negatives (FN), and true negatives (TN). TPs refer to identifiers proposed by the model that either exactly match or have a valid relationship with the human reference identifiers, including cases where a valid identifier is proposed for a label without a corresponding reference. FPs are identifiers that do not match or have an invalid relationship with the human reference, also covering instances where an invalid identifier is proposed in the absence of a reference. FNs correspond to labels for which the model indicates that there is no suitable identifier despite one existing in the human reference, and TNs are identifiers not proposed by either the model or the research team.

Then, we calculated standardized metrics such as precision, recall, F1-score, and accuracy, which are defined next.

• The **precision** of the model is determined by the proportion of identifiers proposed by the model of interest that are correct, assuming that the identifiers from the existing human reference annotations are correct.
  (1) $$\text{Precision}=\frac{\text{TP}}{\text{TP}+\text{FP}}$$
• The **recall** is defined as the proportion of existing identifiers that the model is capable of detecting in each ontology for each type of concept associated with the labels.
  (2) $$\text{Recall}=\frac{\text{TP}}{\text{TP}+\text{FN}}$$
• The **F1-score** metric is the harmonic mean between the precision and recall of the model.
  (3) $$\text{F1-score}=\frac{2\text{TP}}{2\text{TP}+\text{FP}+\text{FN}}$$
• **Accuracy** measures the proportion of correctly classified instances among all instances.
  (4) $$\text{Accuracy}=\frac{\text{TP + TN}}{\text{TP}+\text{FP}+\text{TN}+\text{FN}}$$

To ensure consistency, the same subset of test data is used across all models under study.

In addition, the training process of fine-tuned models was studied through their **learning curves**. This analysis allowed for the acquisition of valuable insights into their ability to learn from data and generalize effectively. The calculation of these metrics was performed using the validation data. The comprehension of these learning curves is imperative for the optimization of model training, scaling, and deployment strategies. Learning curves quantitatively represent the model's learning progression throughout the training process [37]:

• **Training accuracy over time**: Train accuracy refers to the performance metric calculated on the training dataset during the fine-tuning process using the validation data. In this context, accuracy is defined as the proportion of training examples that the model correctly predicts. This performance metric provides a quantitative illustration of the model's performance evolution over time. An increase in this metric over time suggests that the model is acquiring experience and enhancing its learning capabilities.
• **Validation metrics**: The full validation loss and full validation token accuracy are the most reliable metrics for assessing the overall performance of the fine-tuned model. These metrics function as a form of internal validation, ensuring that the training process is proceeding as expected, with a decrease in loss and an improvement in token accuracy.
• **Train and Validation loss**: The training loss is indicative of the model's adaptability to the training data, while the validation loss provides insight into the model's performance on unseen data.

The annotations proposed by an LLM were then subjected to analysis according to the type of concept associated with each label. This included the classification of cell lines, cell types, anatomical structures, and the absence of a specific concept (Table 4). Since each ontology is associated with a specific domain, the importance of the annotations to each ontology depends on the type of concept:

• **Cell line** (CL): The **CLO** and **BTO** ontologies are prioritized since both contain unique identifiers for specific cell lines.
• **Cell type** (CT): The **CL** and **BTO** ontologies are prioritized since both contain unique identifiers for specific cell types.
• **Anatomical structure** (A): The **UBERON** and **BTO** ontologies are prioritized since both contain unique identifiers for specific anatomical structures.
• **Label without a type**: All four ontologies are equally important.

**Table 4:** Number of data per concept type associated with the label in the test data. CL: Cell lines, CT: Cell types, A: Anatomical structures, No concept: label without type of concept.

Type of concept	Number of labels
CL	918
CT	696
A	208
No concept	58
**Total**	**1880**

**Fig. 1:**
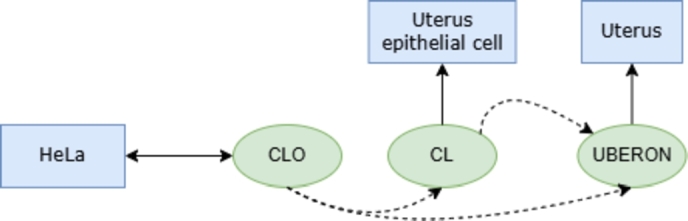
Example of identifier inference.

Furthermore, for each type of concept associated with the label, not only the most relevant ontologies will be taken into account but also those ontologies from which identifiers can be inferred from the primary ones. To illustrate this process, let us consider the cell line “HeLa”. It could be inferred from the CLO identifier that the cell type is “uterine epithelial cell” with its corresponding CL identifier, and that the anatomical structure is the “uterus”, with its corresponding UBERON identifier. Conversely, beginning with the identifier for the cell type “uterine epithelial cell”, it can be inferred that the anatomical structure is the “uterus”. However, the assumption that it corresponds to a HeLa cell line is erroneous, as it may also be consistent with a healthy line (see Fig. 1). In other words, while a hierarchically higher-level concept may be inferred, it is not possible to infer one that implies finer granularity than the one of the starting concept. These inferences are used for calculating the precision of the fine-tuned model for each ontology according to the type of concept associated with the label. Thus, for cell line labels, identifiers for the CLO, CL, UBERON, and BTO ontologies can be obtained; for cell type labels, identifiers for the CL, UBERON, and BTO ontologies will be obtained; and for anatomical structure labels, identifiers for the UBERON and BTO ontologies will be obtained.

In light of the potential inference scenarios previously delineated, the model's efficacy in generating identifiers is appraised on an individual basis, considering the distinct characteristics of each concept and ontology. Additionally, other metrics are calculated to assess the suitability of the obtained identifiers. In this case, the precision calculation includes the **perfect match ratio**, which takes into account the most relevant ontologies for each type of concept associated with the label. For example, in the case of the labels with type of concept “cell line”, a perfect match occurs when the model correctly provides identifiers for both the CLO and BTO ontologies. Thus, the perfect match ratio is calculated by dividing the number of labels that have appropriate identifiers for the two priority ontologies (CLO and BTO) by the total number of labels of that type.

(5) $$\text{Perfect match ratio}=\frac{\text{Number of perfect matches}}{\text{Total labels of type x}}$$

For labels lacking a specified type, the calculation was limited to precision. This approach was adopted due to the study's emphasis on potential error cases, wherein the priority ontologies for each concept type are given due consideration. In the context of labels devoid of a particular concept type, all ontologies are assigned equal importance. For the remaining labels with a defined type, accuracy, recall and F1-score were evaluated alongside precision.

Subsequently, a series of error cases were examined, encompassing scenarios where the identifier generated by the model does not correspond to the reference identifier. However, it should be noted that this discrepancy does not necessarily imply the invalidity of the identifier proposed by the model. This topic will be further elaborated upon in the subsequent sections of this article. This process involves the following steps: (1) searching for the class name for each identifier, (2) searching for and evaluating a common pattern, (3) studying the contributions of the model to the annotation, and (4) checking the errors of the model.

• **Searching for the class name for each identifier:** Once the annotations were obtained and separated by the type of concept associated with the label, the subsequent step was to obtain the class name represented by each identifier. This process enables the assessment of whether the proposed identifier, despite its lack of exact identity with the reference identifier, exhibits a suitable relationship with the reference identifier, thereby qualifying it as a TP. The process of obtaining the class names was carried out using the BioPortal API [38], through which different information can be obtained from the identifier of a particular ontology, including the name of the class it represents.
• **Search and evaluation of a common pattern**: Once the class name for each identifier was obtained, the next phase involved searching for a common pattern between the class name assigned by the model and that of the reference identifier. To this end, a comparative analysis was conducted on the class names produced by the model and the human reference identifier. This analysis was undertaken to assess the textual similarity between the two sets of class names. Following this comparison, a manual review was conducted to ascertain whether the observed similarity indicated a valid relationship between the classes. This approach was adopted to determine the existence of a valid relationship between the identifiers despite them not being identical. A valid relationship occurs when the identifier proposed by the model refers to the same entity but has a different name. For instance, an identifier with the class name “meso-epithelial cell” and the identifier “mesoepithelial cell” are considered equivalent since they represent the same entity with a different name. Another example of a valid relationship is when the model proposes an identifier whose class name corresponds to a hypernym of the class name of the reference identifier, such as the model's identifier corresponding to “breast” and the reference identifier corresponding to “breast epithelium.” In these cases, even though the identifiers are different, they were considered valid and thus a TP. If the relationship is incorrect, it is considered an FP. This pattern search will only be conducted on the most important ontologies for each type of concept associated with the label, as indicated earlier.
• **Contributions of the model to the annotation**: There are instances where the human reference annotations do not have any identifier defined for a given label, but the model is capable of proposing a valid identifier. To conduct this step, a filtering process was implemented, whereby labels with an empty cell for the reference ontology identifier and a populated cell with the model's proposed identifier were identified. Then, the validity of the model's contribution was manually checked. If it is valid, it is considered a TP, and if it is invalid, it is considered as an FP.
• **Errors of the fine-tuned model**: If the model proposes an identifier that is not identical and has no relation to the reference label, it is considered a case of hallucination (FPs).

#### 2.3.2 Human annotation and inter-annotator agreement

A human-centered annotation strategy was employed to assess inter-annotator agreement, with the aim of gaining a clearer understanding of the extent to which errors made by the LLM may stem from inherent ambiguity that even human experts cannot consistently resolve. An additional domain expert, formally trained in ontology engineering, independently annotated a curated set of 50 representative biomedical labels. These labels were stratified into three semantically distinct categories—20 cell lines, 20 cell types, and 10 anatomical structures—and were randomly selected within each category. The expert mapped the 50 labels to four widely used biomedical ontologies: CLO, CL, UBERON, and BTO. Therefore, we had two human annotations: the gold standard and the one from this additional expert. We quantified inter-annotator consistency by computing the **Cohen's kappa** [39], a chance-corrected statistic that measures the degree of agreement between two raters on categorical labels. Kappa values range from -1 (total disagreement) to +1 (perfect agreement), with 0 indicating agreement equivalent to chance. It is defined as:

$$\kappa =\frac{p_{o}-p_{e}}{1-p_{e}}$$

where:

• *κ*: Cohen's kappa coefficient
• $p_{o}$: proportion of observed agreement
• $p_{e}$: proportion of agreement expected by chance

In addition to measuring exact matches via kappa, we introduced a complementary metric, **Soft Agreement**, to capture near-matches that reflect semantic similarity. Specifically, we utilized sentence-transformer embeddings (“all-MiniLM-L6-v2” model) to compute the cosine similarity between the textual labels assigned by each annotator. A soft match was defined as any pair of labels exceeding a similarity threshold of 0.9.

The semantic similarity between two textual inputs *A* and *B* is computed using the cosine similarity of their respective embedding vectors:

$$\text{sim}(A,B)=\frac{\overset{\to }{A}\cdot \overset{\to }{B}}{\left\|\overset{\to }{A}\|\cdot \|\overset{\to }{B}\right\|}$$

where $\overset{\to }{A}$ and $\overset{\to }{B}$ are the embeddings generated by a pre-trained language model, and ‖⋅‖ denotes the Euclidean norm. This measure captures the angular similarity between the vectors, effectively quantifying the semantic closeness of the two texts and enabling the assessment of agreement even in cases where annotators selected ontologically related but non-identical terms (e.g., “heart” vs. “left ventricle”).

This dual-method approach facilitates a more nuanced assessment of agreement by accounting for both strict categorical concordance and flexible semantic overlap.

#### 2.3.3 Comparison with the text2term tool

The objective of this experiment is to compare the performance of the LLM with a tool that applies traditional methods for the annotation task. For this task, we used the text2term tool [14] as a state-of-the-art reference. Consequently, the labels of the test set were annotated with this tool.

We selected the text2term tool because it has demonstrated high precision in annotating biological entities compared to human-verified benchmarks, significantly reducing the need for manual curation. Notably, the text2term tool is a subject of ongoing development and refinement, a practice that has been shown to enhance its efficacy in standardizing biomedical metadata. In addition, we selected text2term for comparison with our model because it integrates both BioPortal [38] and Zooma [40], two reference tools in the annotation task.

## 3 Results

The data used in this article and the results obtained are available at https://github.com/tecnomod-um/BiosamplesLLMAnnotation.

### 3.1 Validation of the fine-tuned models

In this section, the learning curves of each of the fine-tuned models are analyzed (see Supplementary Figure 1):

• **Training accuracy over time**: The training accuracy increases with the number of steps, ranging between 80% and 98% in all three models. The figure illustrates the model's performance increasing over time, indicating that the model is gaining experience and improving its learning ability.
• **Validation metrics**: The loss is expected to decrease and token accuracy to improve, which is precisely what occurs in all three models.
• **Train and Validation loss**: Using a number of epochs equal to 6, both training and validation losses decrease. This equilibrium suggests that the models are well-trained and can effectively generalize the knowledge learned.

All three models, the fine-tuned GPT-3.5, the fine-tuned GPT-4o, and the fine-tuned GPT-4o-mini, demonstrate good training performance throughout the fine-tuning process, as indicated by their learning curves.

### 3.2 Performance of the models

**Fig. 2:**
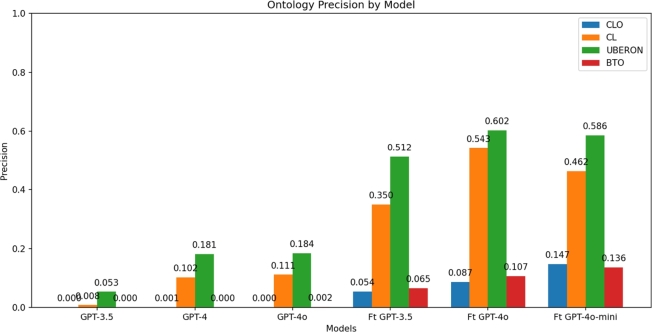
Comparison of the precision of the models studied.

Fig. 2 shows the precision of the models for each of the four ontologies with the same test data. The fine-tuned GPT-4o model demonstrated a range of precision outcomes, varying from 8.7% to 61% across the different ontologies. In comparison, the fine-tuned GPT-3.5 model achieved precision rates ranging from 5.4% to 52%, and the fine-tuned GPT-4o-mini model achieved precision rates ranging from 14.7% to 59%. Conversely, the precision of the base models did not reach 20%, with the precision being close to 0% in several cases. Instances where the model's output did not conform to the expected format were disregarded in the subsequent analysis. The base GPT-3.5 model exhibited four instances of these errors, the base GPT-4 model had one, the fine-tuned GPT-3.5 model showed two, the fine-tuned GPT-4o model showed 436, and the fine-tuned GPT-4o-mini model displayed 149 formatting errors.

In this case, the fine-tuned GPT-4o model demonstrated a slightly better performance on the CL and UBERON ontologies but produced a higher number of formatting errors (436 out of 1880 labels). In contrast, the fine-tuned GPT-4o-mini model achieved nearly equivalent precision while significantly reducing the number of formatting errors (149 out of 1880). Therefore, the fine-tuned GPT-4o-mini model was selected for a detailed analysis of its performance, due to its optimal balance of accuracy, efficiency, and cost-effectiveness.

Regarding the experiment in which the training data included label descriptions, as shown in Supplementary Figure 2, the precision of the model in the annotation task decreased. Therefore, the provision of label descriptions as supplementary context led to an augmented prevalence of hallucinations.

### 3.3 Detailed performance of the fine-tuned GPT-4o-mini model

Table 5, Table 6, Table 7 show the results by type of concept associated with the labels. In those tables, there are three possible origins for TPs: (I) the identifier proposed by the fine-tuned model is identical to the reference; (R) the identifier proposed by the fine-tuned model is not identical to the reference, but shows a valid relationship with the reference identifier; and (C) when the fine-tuned model proposes a valid identifier for a label in the absence of a corresponding human reference identifier. The FPs emerge from two distinct scenarios: (1) cases where the fine-tuned model proposed identifiers that either lack any relationship with the reference identifier or have an incorrect relationship, both being classified as model errors (E); and (2) cases where the fine-tuned model proposed an identifier that does not have a corresponding human-assigned reference identifier, categorized as incorrect contribution (IC).

**Table 5:** Table of results for the fine-tuned GPT-4o-mini for labels with type of concept “cell line”.

TP			FP		FN	TN	Precision	Recall	F1-score	Ontologies
I	R	C	E	IC						
65	1	4	316	83	114	221	0.149	0.380	0.214	**CLO**
409	0	1	323	15	54	2	0.548	0.884	0.677	**CL**
488	0	2	270	4	27	13	0.641	0.948	0.765	**UBERON**
55	0	7	244	76	122	300	0.162	0.337	0.219	**BTO**

**Table 6:** Table of results for the fine-tuned GPT-4o-mini for labels with type of concept “cell type”.

TP			FP		FN	TN	Precision	Recall	F1-score	Ontologies
I	R	C	E	IC						
282	26	4	333	9	16	3	0.477	0.951	0.635	**CL**
415	0	0	237	4	14	3	0.633	0.967	0.765	**UBERON**
62	1	5	256	84	64	201	0.167	0.515	0.252	**BTO**

**Table 7:** Table of results for the fine-tuned GPT-4o-mini for labels with type of concept “anatomical structure”.

TP			FP		FN	TN	Precision	Recall	F1-score	Ontologies
I	R	C	E	IC						
84	4	0	109	0	1	0	0.447	0.989	0.615	**UBERON**
17	0	1	115	19	18	28	0.118	0.500	0.191	**BTO**

Table 5 presents the results obtained for the cell line labels. It is noteworthy that there is a single instance of non-identical identifiers that exhibit a valid relationship. This finding suggests that, in general, when the fine-tuned model proposes a different identifier, it tends to be incorrect as it does not maintain a valid relationship with the human-provided reference identifier. Furthermore, the fine-tuned model predominantly exhibits invalid contributions, with the CLO ontology demonstrating the highest prevalence of valid contributions. However, the proportion of TNs increases for CLO and BTO ontologies. In these cases, the model identifies instances when there is an absence of suitable identifiers for the label within those ontologies.

Table 6, Table 7 present the results for cell type and anatomical structure labels, respectively. As was the case in the preceding instance, the majority of the contributions are invalid. Whenever the fine-tuned model suggests an alternative identifier, it is usually incorrect. In these cases, the BTO ontology also has the highest number of TNs compared to the other ontologies.

Regarding errors, we observed that when the model fails, it typically generates random identifiers, some of which may not correspond to existing ontology terms, or retains placeholder identifiers such as “CLO_0000000” as shown in the example output format (“[CLO_0000000, CL_0000000, UBERON_0000000, BTO_0000000]”). Nonetheless, even when the predicted identifiers are incorrect, the LLMs consistently adhere to the expected format.

#### 3.3.1 Performance by type of concept associated with the label

**Fig. 3:**
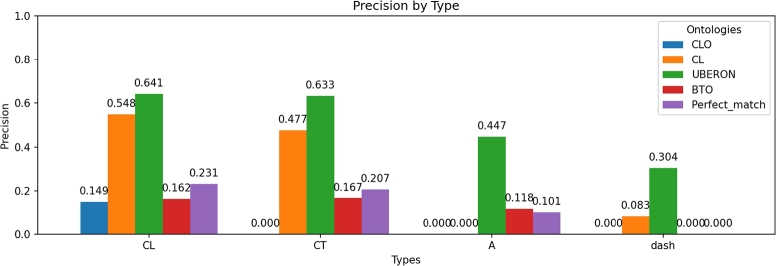
Precision of the fine-tuned model for each ontology according to the type of concept associated with the label. The purple bar represents the perfect match ratio for each of the types of concepts. That is, when the label is annotated with the same real-world entity in the specific ontology (CLO, CL, UBERON, and BTO).

The precision of the fine-tuned GPT-4o-mini model was calculated for each ontology according to the type of concept associated with the label (see Fig. 3). Precision ranged for cell lines from 14% to 65%, showing higher precision for the CL and UBERON ontologies but lower precision for the CLO and BTO ontologies. The low precision observed for the CLO and BTO ontologies is due to the complexity introduced by alphanumeric cell line labels, which makes annotation more challenging. With regard to the perfect match ratio, which refers to correctly identifying both CLO and BTO identifiers, the success rate was 23%. However, as previously mentioned, the precision for CLO was relatively low, making it challenging to achieve a perfect match with the BTO identifier.

Precision values for cell types ranged from 16% to 64%, again favoring UBERON and CL over BTO. The observed low precision for the BTO ontology can be attributed to its broad scope, which includes identifiers for cell lines, cell types, and anatomical structures. This extensive scope introduces ambiguity, leading to erroneous identifier assignment to labels. In this case, the perfect match ratio decreased slightly compared to the previous type of concept.

Anatomical structures showed lower precision, 44.7% for UBERON and 11.8% for BTO. The perfect match ratio for this type of concept was around 10%, which indicates a decline in correct identifiers for both UBERON and BTO in comparison to prior studies.

The labels without a concept type (dash in Fig. 3) exhibited precision between 8.3% and 30.4%, meaning that the absence of clearly defined concept types makes the annotation task more difficult.

**Fig. 4:**
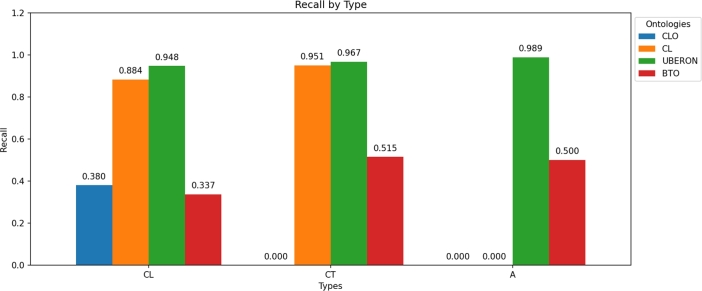
Recall of the fine-tuned model for each ontology according to the type of concept associated with the label.

The recall values (see Fig. 4) for the three concept categories analyzed (cell line, cell type, and anatomical structure) ranged from 33.7% to 98.9%, with the highest recall observed for the CL and UBERON ontologies and the lowest one for the CLO and BTO ontologies. This exceptionally high recall rate for the CL and UBERON ontologies indicates that the model is highly effective at capturing nearly all relevant identifiers, thereby minimizing the risk of missing important data points. Additionally, the high recall observed across all concept types underscores the model's capacity to consistently support applications that rely on precise identifier detection. Conversely, the low recall observed for the BTO ontology can be attributed to its extensive array of identifiers, which confuse the model. The low recall seen in the CLO ontology is likely due to the complexity introduced by its alphanumeric labels.

**Fig. 5:**
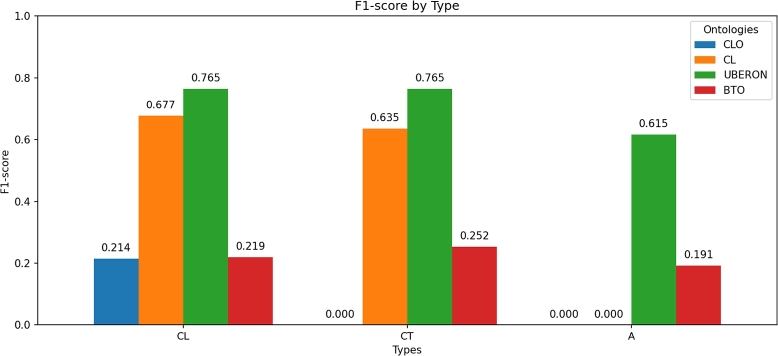
F1-score of the fine-tuned model for each ontology according to the type of concept associated with the label.

The F1-score metric of the fine-tuned model was calculated for each ontology according to the type of concept associated with the label (Fig. 5). The CLO (in cell lines) and BTO ontologies exhibited the lowest F1-scores for the three concept types analyzed, while CL and UBERON achieved the highest. These findings are consistent with the precision and recall analysis. The observed variation in F1-scores across ontologies indicates that certain ontology structures inherently pose greater complexity for the model, highlighting opportunities for further refinement and targeted training to improve overall performance.

### 3.4 Human annotation and inter-annotator agreement

#### 3.4.1 Inter-annotator agreement

In this section, we study the agreement between an expert and the gold standard to identify which ontologies are more challenging from the annotation perspective. The annotations provided by the two human annotators involved in the study are compared: the gold standard annotator used to evaluate the annotation performance of the fine-tuned model, and the additional expert annotator incorporated subsequently.

As summarized in Table 8, the highest agreement was observed for the CLO ontology, with a Cohen's Kappa score of 0.715 (substantial agreement) and a soft semantic agreement of 0.9, indicating both high literal concordance and strong conceptual similarity in the selected entities. Similarly, the BTO ontology demonstrated substantial agreement, with a soft agreement score of 0.62 and a Kappa value of 0.509 (moderate agreement).

**Table 8:** Comparison of Soft Agreement and Cohen's Kappa for the additional expert and human gold standard.

Ontology	Soft Agreement	Cohen's Kappa (Exact Match)
CLO	0.900	0.715
CL	0.340	0.293
UBERON	0.420	0.411
BTO	0.620	0.509

In contrast, the agreement was notably lower for the CL ontology, which yielded the lowest scores among the four categories, with a soft agreement of 0.34 and a Kappa score of 0.293 (fair agreement). These results potentially reflect ambiguity or overlap in the conceptual boundaries of entities within the CL ontology, i.e., being an ontology with a high degree of granularity, human annotators can differ in the level of cell type specificity. In consequence, it may have led to divergent interpretations. The UBERON ontology yielded intermediate agreement levels, with a soft score of 0.42 and a Kappa score of 0.411 (moderate agreement).

#### 3.4.2 Revision of the performance of the model

The previous results show that the human annotators exhibited a higher level of agreement with CLO and BTO ontologies, possibly due to the structure, clarity, or granularity of these ontologies. Thus, the entities of these ontologies may align more closely with the expectations and decision-making processes of expert annotators. For these ontologies, the human annotation achieved higher precision levels than the fine-tuned model. We consider that the errors made by the fine-tuned model for these ontologies may stem from its inadequacy in interpreting the alphanumeric codes associated with cell lines (Fig. 3). In the case of BTO, these errors may also be attributed to the extensive diversity of terms encompassed within the ontology, as previously discussed.

**Fig. 6:**
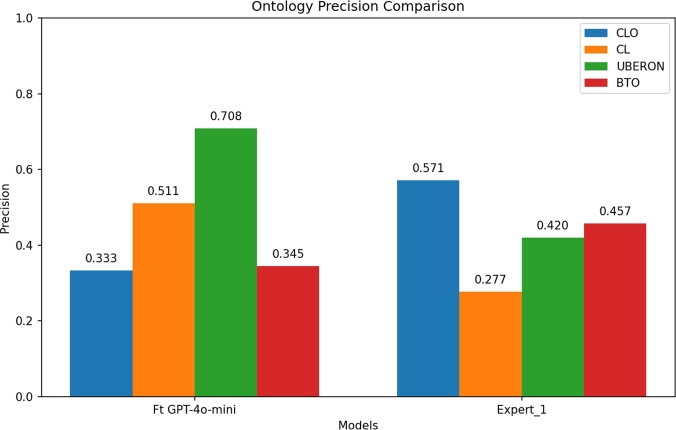
Precision comparison between the fine-tuned model and the human expert.

Contrariwise, the precision of the fine-tuned GPT-4o-mini model was higher for CL and UBERON (see Fig. 6) for the 50 randomly selected labels. The inter-annotator agreement among humans was lower for these ontologies due to the fine-grained and overlapping nature of the concepts within both ontologies, as well as the ambiguity of the labels. However, the fine-tuned model was able to effectively mitigate these challenges by being trained to consistently assign specific identifiers, thereby reducing annotation variability.

As illustrated in Table 9, a comparison of the processing time required by a fine-tuned model and the additional human expert for completing the same annotation task is presented. The fine-tuned model has been shown to significantly outperform the human annotator in terms of efficiency, with an average time of 1.23 seconds per annotation. In contrast, Human Expert 1 required 420 minutes (504 seconds per annotation).

**Table 9:** Comparison of processing time.

Model	Total Time (min)	Time per Annotation (s)
Fine-tuned model	1	1.23
Human Expert 1	420	504

### 3.5 Comparison with the text2term tool

**Fig. 7:**
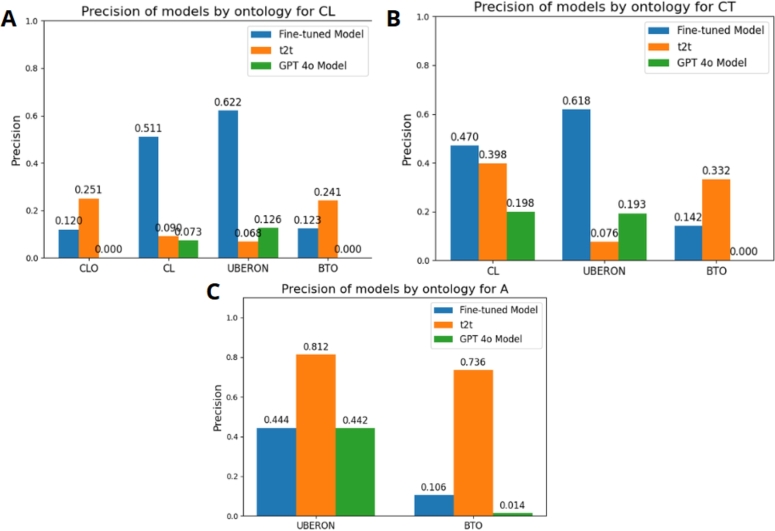
Precision comparison of the GPT-4o model, the fine-tuned model, and text2term tool for each ontology according to the type of concept associated with the label. A) Precision comparison for each ontology in the data with labels with the type of concept “cell line”. B) Precision comparison for each ontology in the data with labels with the type of concept “cell type”. C) Precision comparison for each ontology in the data with labels with the type of concept “anatomical structure”.

Fig. 7 shows the precision obtained for cell lines (A), cell types (B), and anatomical structures (C) using the text2term tool, the GPT-4o base model, and the fine-tuned GPT-4o-mini model.

The results indicate that text2term exhibits superior performance in identifying terms related to anatomical structures, with precision ranging from 73.6% to 81.2%. This significantly surpasses the performance of both the base GPT-4o model (precision ranging from 1.4% to 44.2%) and its fine-tuned version (precision ranging from 10.6% to 44.4%). However, for cell lines and cell types, the fine-tuned model outperforms both text2term and the base GPT-4o model. It achieves precision rates ranging from 47% to 51% for the CL ontology and from 61% to 63% for the UBERON ontology. In contrast, text2term reaches precision rates of only 0.9% to 40% for the CLO ontology and 6.8% to 7.6% for the UBERON ontology. Nonetheless, text2term has demonstrated superior precision rates for both CLO and BTO ontologies when annotating cell line labels, as well as for the BTO ontology in the annotation of cell type labels. This demonstrates that while the fine-tuned model can infer the cell type and anatomical structure associated with a given cell line or cell type, the text2term tool merely retrieves an identifier for the provided label without performing any inference. It is noteworthy that the number of labels associated with anatomical structures was lower than with cell lines or cell types. Consequently, in this particular case study, the refined model demonstrated a superior mean precision in the annotation task across the four selected ontologies in comparison to the text2term tool. In terms of execution times, the fine-tuned model requires 49 minutes and 12 seconds to annotate the 1,880 biological labels, whereas text2term completes the task in 7 minutes and 3 seconds. When examining the execution time per annotation, the fine-tuned model requires approximately 1.56 seconds, while the text2term tool completes each annotation in about 0.23 seconds.

## 4 Discussion

This work explores the application of LLMs to expedite the semantic integration of data by augmenting the automation of annotating biological data to ontologies. This task is of paramount importance due to the fact that biological databases frequently employ labels that vary between repositories, even when the entities in question are identical in reality. This discrepancy impedes the integration and utilization of biological information, a field that necessitates an understanding of the relationships between multiple and heterogeneous biological entities. First, the performance of several models was evaluated: OpenAI's GPT-3.5, GPT-4, and GPT-4o, as well as the fine-tuned models developed in this project based on GPT-3.5, GPT-4o, and GPT-4o-mini, all tested with the same dataset (see Fig. 2). The evaluation revealed that the fine-tuned models exhibited superior performance in comparison to the base models, which demonstrated a low precision level that substantiates the utilization of trained models for this particular task. This finding underscores the efficacy of the fine-tuning process applied to GPT-3.5, GPT-4o, and GPT-4o-mini. The process has led to a substantial enhancement in the model's capacity to accurately identify an appropriate identifier for a given label, notwithstanding the variations among labels.

The enhancement in precision is attributed to the model learning specific features and patterns from the data domain, which reduces errors and improves prediction accuracy. In essence, fine-tuning empowers the model to adapt more effectively to particular contexts, a capability that is indispensable for applications within specialized domains and tasks.

However, GPT-4o has shown marginal superiority in precision for certain ontologies; nevertheless, it exhibited a higher frequency of formatting errors. The GPT-4o-mini version exhibited comparable precision with a reduced number of errors, thus becoming the preferred model.

Despite its affordability, the GPT-3.5 base model exhibits suboptimal precision, rendering it ill-suited for the annotation task. The same applies to the GPT-4 and GPT-4o base models: despite their relatively low costs, their precision remains insufficient, though slightly better than GPT-3.5. The fine-tuning process is more expensive because it involves the development of the model itself. However, the fine-tuned models exhibit a marked enhancement in precision, surpassing the performance of the base models, with the fine-tuned GPT-4o-mini model demonstrating the most notable advancement. This fine-tuned model is deemed suitable for the label annotation task, thereby validating the cost-effectiveness of the investment in the fine-tuning process over time.

In addition, incorporating label descriptions into the training data to provide supplementary context resulted in a decline in annotation precision and an increase in hallucinations, as illustrated in Supplementary Figure 2. We hypothesize that this effect is due to the introduction of noise and conflicting signals within the additional text, which impaired model performance rather than enhancing it. This observation aligns with recent findings emphasizing that the quality and relevance of contextual information often outweigh its sheer volume in large language model performance [41]. Furthermore, similar to the findings of other studies [42], we observed that targeted context pruning improved task precision compared to supplying full record content. These results suggest that, even when technical limitations on input size are lifted, careful curation of context remains critical to optimize model behavior.

An evaluation of the performance of the fine-tuned GPT-4o-mini model was then carried out, where the model's precision was calculated for each of the ontologies according to the type of concept associated with the label.

The precision for the “cell line” concept type (see Fig. 3) is notably high for both the CL and UBERON ontologies. In contrast, the CLO ontology shows the lowest precision, despite its strong relevance to this concept type. This discrepancy may be attributed to the fact that cell line labels frequently consist of alphanumeric codes, which pose a challenge for the model in establishing a clear relationship between the label and its identifier. As a result, precision decreases for the CLO ontology, which specifically focuses on cell lines. Furthermore, in certain instances, the original labels retrieved from databases may include not only the name of the cell line but also descriptive information, such as the tissue or organ of origin (example in Table 2). The presence of these descriptive words helps the fine-tuned model to suggest an identifier for CLO in the case of cell lines, but also to infer the corresponding cell type of CL ontology and the anatomical structures of UBERON (see Table 10). The encoding of label names also affects the identification of appropriate relations between non-identical identifiers, as no appropriately related non-identical identifier to the reference identifier was found in three of the four ontologies under study (TP, R) (see Table 5).

**Table 10:** Example of coded and descriptive labels for cell lines.

Biosample label	Type of concept	CLO_Control	CLO_Model
697	CL	CLO_0001008	CLO_0003837
HS-SY-II	CL	CLO_0050072	CLO_0004001
22RV1_prostate_carcinoma	CL	CLO_0001200	CLO_0001200

As was the case in the preceding instance, the highest precision for the “cell type” concept (see Fig. 3) was ascertained to be in the UBERON and CL ontologies, whereas the BTO ontology demonstrated the least precision.

The precision for the “anatomical structure” concept type labels (see Fig. 3) for the UBERON and BTO ontologies was 44.7% and 11.8%, respectively. This was the most challenging type of concept for the fine-tuned model, perhaps because the number of labels of concept type “anatomical structure” was lower in both the training and test sets compared to “cell lines” and “cell types”.

The model demonstrated a high degree of success, achieving perfect match rates of over 10-23% for the majority of concept types. However, it exhibited challenges with specific ontologies and less prevalent concept types, underscoring difficulties in label-identifier relationships and the significance of descriptive labeling.

Lastly, the precision of those labels that do not present a concept type was studied and indicated as “dash” (see Fig. 3), and it ranged from 0% to 30.4%, with the CL and UBERON ontologies showing the highest precision. This phenomenon can be attributed, once again, to the predominance of numerical and alphabetic characters in the labels lacking a concept type.

Regarding recall across the three concept types associated with the labels under study, it is notably high for both the CL and UBERON ontologies. This finding underscores the challenges posed by the alphanumeric codes employed in cell line entities, which complicate the annotation process. The BTO ontology, with its extensive array of identifiers, further exacerbates the complexity of the task (see Fig. 4).

In relation to the F1-*score* (see Fig. 5), in the case of the cell line concept type, the lowest percentage was observed for the CLO ontology and the highest for UBERON, which is consistent with the explanation for precision.

Overall, the fine-tuned GPT-4o-mini model achieved F1-score percentages ranging from 19% to 77% across the different ontologies. The findings suggest that the model demonstrates optimal performance for the CL and UBERON ontologies in the annotation task, while encountering challenges when employed with the CLO and BTO ontologies.

Finally, accuracy results are presented only in the supplementary material (see Supplementary Figure 3). They do not offer any additional or meaningful insights, but reinforce the conclusions drawn from the other metrics.

Concerning the comparison of our approach with text2term, we highlight that text2term is a rule-based tool that relies primarily on traditional information retrieval techniques like TF-IDF and string similarity metrics, comparing input terms directly with ontology labels and synonyms. It does not employ machine learning techniques; rather, it matches based on lexical overlap and frequency patterns within curated ontologies. In contrast, the fine-tuned GPT-4o-mini model is a deep learning-based transformer trained on large-scale textual data and fine-tuned for specific biomedical annotation tasks. It understands context, semantics, and even obscure or abbreviated terms by leveraging pre-trained language representations. This ability to generalize beyond surface-level patterns enables the model to adapt to entity types with limited or ambiguous string features, such as cell lines.

The performance comparison with the text2term tool revealed that text2term excels in identifying anatomical structure terms, exhibiting higher precision than both the base GPT-4o model and fine-tuned GPT-4o-mini model. However, for cell lines and cell types, the fine-tuned model outperforms both, particularly with the CL and UBERON ontologies. This limitation arises from text2term's approach of searching for appropriate terms in alternative ontologies when a suitable match cannot be found within the specified ontology. However, text2term still achieves higher precision for CLO and BTO ontologies in certain cell line and cell type annotations, although it lacks inference capabilities.

The divergence in performance between text2term and the fine-tuned GPT-4o-mini model can be attributed to the fundamental differences in their architectures and the nature of the entity types they process. Text2term's superior precision on anatomical structures can be attributed to its reliance on TF-IDF-based matching, which benefits from the rich semantic coverage, hierarchical structure, and extensive synonym annotations found in anatomical ontologies like UBERON. These labels tend to be linguistically descriptive (e.g., “breast epithelium”), making them well-suited for text-based similarity approaches. In contrast, cell lines and cell types often use alphanumeric identifiers (e.g., “HEK293”, “A549”) or abbreviations that lack semantic content and are poorly handled by term-frequency methods. The fine-tuned GPT-4o-mini model, trained on contextual data, excels in these cases because it can infer meaning and associations beyond surface string similarity, leveraging learned representations to resolve such opaque labels. This observation underscores the fact that text2term is optimized for semantically rich, language-based terms, whereas fine-tuned GPT-4o-mini exhibits superior proficiency in interpreting sparse or coded biological entities. Therefore, when the objective is to annotate a label related to cell lines or cell types within a specific ontology of interest, the fine-tuned model outperforms the text2term tool due to its ability to infer contextual information. Conversely, if the goal is limited to mapping a label to any ontology without the need for inference, the annotation provided by text2term may still constitute a viable alternative.

Regarding execution times, the fine-tuned model is significantly slower, taking nearly 50 minutes to annotate all 1880 labels compared to text2term's 7 minutes. Although the fine-tuned model is generally slower than the text2term tool in terms of total execution time, the time required per annotation is not excessively high. Specifically, the fine-tuned model requires approximately 1.56 seconds per annotation, whereas text2term requires 0.23 seconds. This suggests that, despite its overall longer processing time, the fine-tuned model remains a viable option for annotation tasks, particularly when higher precision and inference capabilities are required.

To evaluate the consistency of annotations, we employed a human-centered approach supported by a two-pronged assessment methodology. In summary, the inter-annotator agreement analysis reveals a consistent ontology-dependent pattern in annotation reliability. CLO and BTO exhibit the highest levels of concordance across the expert comparison, with moderate-substantial agreement in both exact and semantically similar term selections. These results suggest that the structure, clarity, or granularity of these ontologies may align more closely with expert annotators' expectations and decision-making processes. Conversely, the lower agreement scores observed for CL and UBERON, highlight persistent challenges in achieving annotation consistency, due to overlapping concept boundaries, the granularity of the terms, and ambiguities in label interpretation.

The comparison between the fine-tuned model and the human expert annotator reveals important insights into the capabilities and limitations of automated annotation in complex biomedical ontologies. The results suggest that the model is particularly well-suited to ontologies characterized by overlapping concept boundaries or terminological ambiguity, where human agreement tends to be lower. This strength likely stems from the model's consistent assignment of trained identifiers, which reduces the variability seen in human annotation. Conversely, for the CLO ontology, the human expert outperformed the model, underscoring the model's limitations in handling ontologies characterized by high lexical complexity. Moreover, in the case of BTO ontology, the human agreement was still strong, suggesting that the model is capable of approaching expert-level performance when concepts are well defined and semantically distinct. Therefore, the model's errors appear to arise primarily from the structural and lexical challenges previously described, rather than from ambiguity. These findings underscore the complementary strengths of human and machine annotators and support the integration of fine-tuned language models into biomedical curation workflows, particularly in tasks requiring scalability, speed, and consistency, while still relying on expert oversight for complex or ambiguous domains.

When comparing the time required to complete the annotation task for the 50 labels, a clear contrast emerges between the fine-tuned model and the human expert. As the results illustrate, the fine-tuned model has been shown to significantly outperform the human annotator in terms of efficiency, with an average time of 1.23 seconds per annotation. These results underscore the substantial efficiency gains offered by the fine-tuned model, highlighting its potential for large-scale annotation tasks where time and scalability are critical factors. While human expertise remains essential for quality assurance and complex cases, the model's speed offers a compelling advantage in practical applications.

One limitation of our work is the necessity for expert participation in the evaluation of the model. This necessity stems from the manual, label-by-label review of results. This was the reason why accuracy, F1-score, and recall metrics were calculated only for the fine-tuned models, as the calculation of these metrics requires considerable manual work. Another limitation is the manual cost required to build the initial dataset, in which each of the labels has been manually mapped to the four ontologies of interest. However, the results obtained show that our trained model can suggest and facilitate the task of the expert regarding new annotations. On the other hand, the exploration of alternative prompting strategies was not undertaken. A constrained output format was deliberately adopted, prohibiting explanatory text and enforcing a structured response, with the objective of facilitating systematic and reproducible evaluation. However, it is acknowledged that the allowance of reasoning traces could potentially improve accuracy, particularly in complex tasks such as ontology alignment.

As future directions for this project, identifying the minimum size of the training dataset that would ensure the precision of the fine-tuned model without compromising its integrity would serve to reduce development costs. While the present study centers on deterministic, one-to-one mappings of biological sample labels to unambiguous ontological identifiers, an important avenue for future research involves exploring scenarios that permit multi-class or probabilistic mappings. Such approaches could offer deeper insights into the inherent ambiguity of biological labeling and support the development of more flexible and robust interoperability frameworks. Additionally, it could be worth investigating the implementation of Retrieval-Augmented Generation (RAG), where the model is provided with files containing ontologies that it can reference when generating a response. It would also be interesting to assess how the fine-tuned model performs with other organisms, such as mice, since the biological samples used for training are all of human origin. Moreover, future research could investigate the trade-offs between constrained and other prompting strategies in terms of performance, interpretability, and automation potential across diverse domains. Finally, it would be interesting to compare the performance of open-source LLMs, although our preliminary results on related problems suggest that their performance would be lower.

## 5 Conclusions

Our research demonstrates that the refinement of large language models can yield tools that outperform existing tools in terms of performance, particularly in labels that align more closely with natural language. Consequently, LLMs emerge as promising instruments for supporting annotation tasks, capable of enhancing the semantics of heterogeneous biological datasets and enabling data interoperability. We consider that our results show that generative artificial intelligence can significantly contribute to increasing the value and usefulness of existing datasets for biomedical researchers. Nevertheless, their current level of performance makes them a good assistant but not a replacement for human data curators.

## CRediT authorship contribution statement

**Andrea Riquelme-García:** Writing – review & editing, Writing – original draft, Software, Methodology, Investigation, Conceptualization. **Juan Mulero-Hernández:** Writing – review & editing, Writing – original draft, Validation, Methodology, Conceptualization. **Jesualdo Tomás Fernández-Breis:** Writing – review & editing, Supervision, Methodology, Investigation, Conceptualization.

## Declaration of Competing Interest

The authors declare that they have no known competing financial interests or personal relationships that could have appeared to influence the work reported in this paper.

## Appendix A Supplementary material

MMC Supplementary table with the information of the databases used and supplementary figures showing additional results. MMC

## Footnotes

1 https://www.snomed.org.

2 https://github.com/tecnomod-um/BiosamplesLLMAnnotation.
