# Supplementary material for: Annotation of biological samples data to standard ontologies with support from large language models
Source: Comput Struct Biotechnol J. 2025 May 26;27:2155–67. doi: 10.1016/j.csbj.2025.05.020 (PMC12162076; doi:10.1016/j.csbj.2025.05.020)
Supplement: MMC — Supplementary table with the information of the databases used and supplementary figures showing additional results. [file mmc1.pdf]

Supplementary material

Databases	URL
dbSUPER	https://asntech.org/dbsuper/data/dbSUPER_SuperEnhancers_hg19.tsv
ENdb	http://www.licpathway.net/ENdb/file/download/ENdb_enhancer.txt
EnDisease	http://health.tsinghua.edu.cn/jianglab/endisease/download_files/endisease.tar.gz
EnhancerDB	http://lcbb.swjtu.edu.cn/EnhancerDB/_download/enhancer.gz
EnsemblReguLatory Build v.109	https://ftp.ensembl.org/pub/release-109/regulation/homo_sapiens/homo_sapiens.GRCh38.Regulatory_Build.regulatory_features.20221007.gff.gz
FANTOM5	https://FANTOM5.gsc.riken.jp/5/datafiles/latest/extra/Enhancers/human_permissive_enhancers_phase_1_and_2.bed.gz
RAEdb	http://www.computationalbiology.cn/RAEdb/download.html
RefSeq Release 110	https://ftp.ncbi.nlm.nih.gov/refseq/H_sapiens/annotation/annotation_releases/110/GCF_000001405.40_GRCh38.p14/GCF_000001405.40_GRCh38.p14_genomic.gff.gz
VISTA Enhancer	https://vista-enhancer.lbl.gov/cgi-bin/imagedb3.pl?search.form=no;search.result=yes;page=1;page_size=20000;form=search;action=search;show=1
CancerEnD	https://webs.iiitd.edu.in/raghava/cancerend/cancerend.csv
JEME	http://yiplab.cse.cuhk.edu.hk/jeme/
FOCS	http://acgt.cs.tau.ac.il/focs/data/
HACER	http://bioinfo.vanderbilt.edu/AE/HACER/download/T1.txt
EnhancerAtlas 2.0	http://www.enhanceratlas.org/downloadv2.php
ChromHMM	http://hgdownload.cse.ucsc.edu/goldenpath/hg19/encodeDCC/wgEncodeBroadHmm
SEA 3.0	http://218.8.241.248:8080/SEA3/download/SEA00101.bed
GenoSTAN	https://www.cmm.in.tum.de/public/paper/GenoSTAN/GenoSTAN_enhancers.bed.gz
SEdb 2.0	https://bio.liclab.net/sedb/download/new/package/SE_package_hg38.bed
scEnhancer	http://enhanceratlas.net/scenhancer/data/download/
EnhFFL	http://lcbb.swjtu.edu.cn/EnhFFL/download/
GeneHancer 4.8	https://genome.ucsc.edu/cgi-bin/hgTables
Roadmap 15-states + DNaseI	https://egg2.wustl.edu/roadmap/data/byDataType/dnase/BED_files_enh/
TiED	http://lcbb.swjtu.edu.cn/TiED/static/files/Active_enhancers_of_10_tissues.xlsx
SCREEN V3	https://downloads.wenglab.org/Registry-V3/GRCh38-cCREs.pELS.bed
DiseaseEnhancer	http://biocc.hrbmu.edu.cn/DiseaseEnhancer/RFunctions/enh2disease-1.0.2.txt
3D Genome Browser	http://3dgenome.fsm.northwestern.edu/downloads/hg38.TAD.zip
TADKB	http://dna.cs.miami.edu/TADKB/download/TAD_annotations.tar.gz

Supplementary Table 1: Databases used to obtain the input data and their corresponding URL.

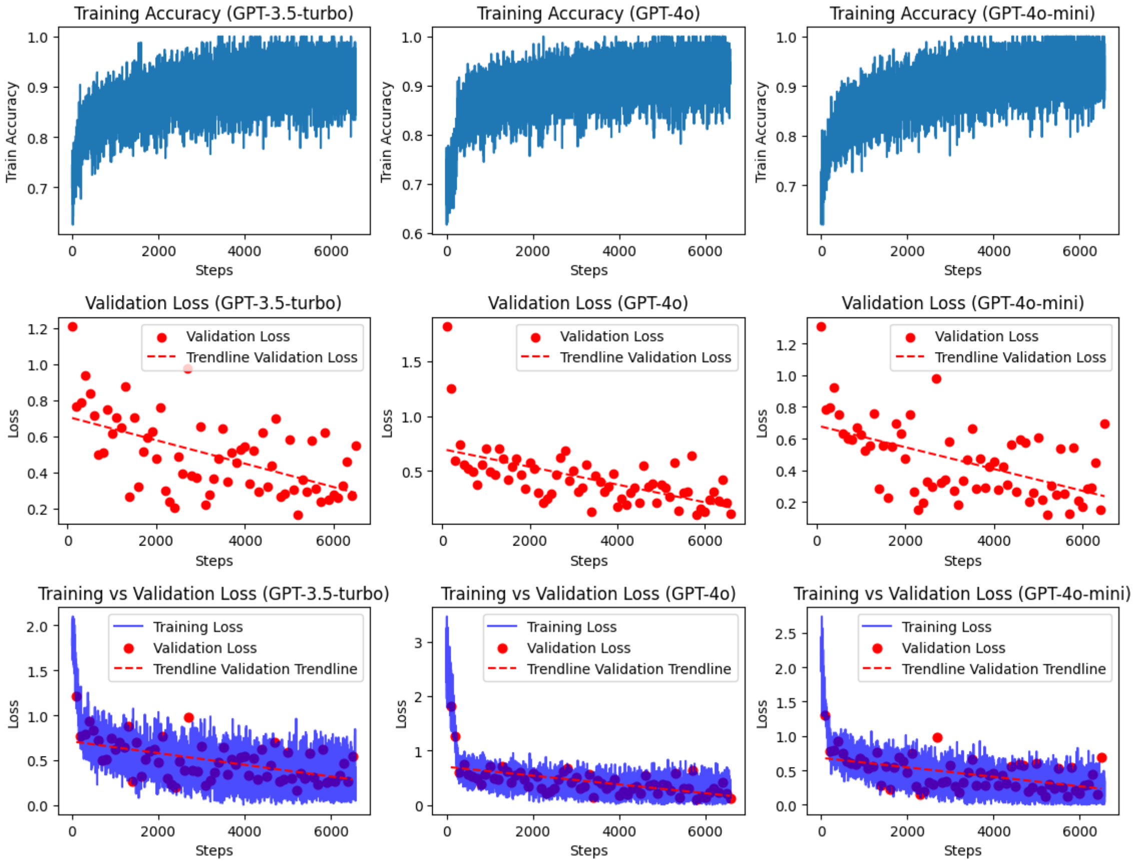

Supplementary Figure 1: Training metrics for the different fine-tuned models.

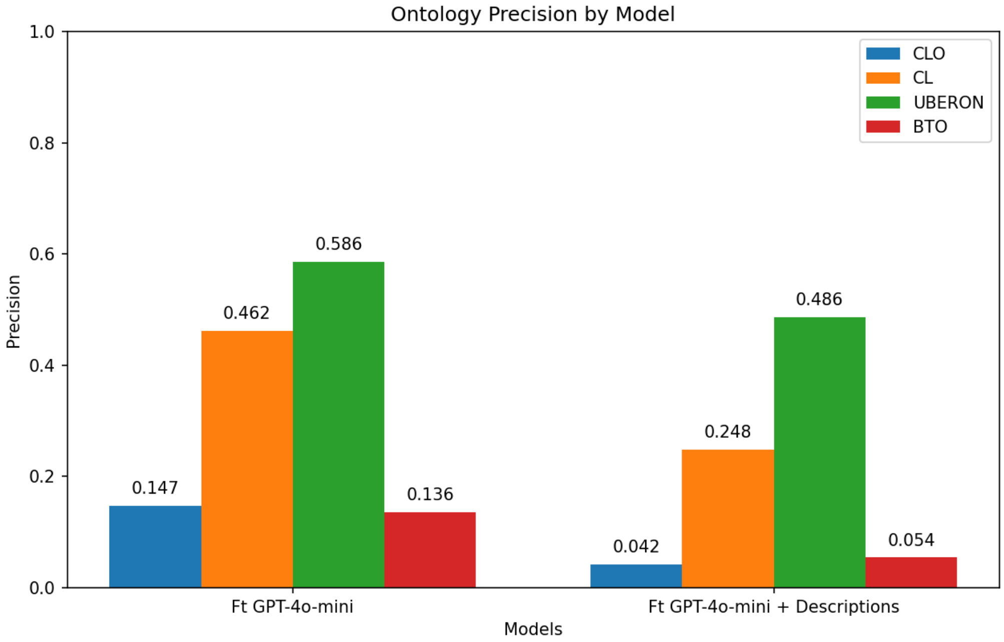

Supplementary Figure 2: Comparison of the performance of the fine-tuned model without and with descriptions of the label.

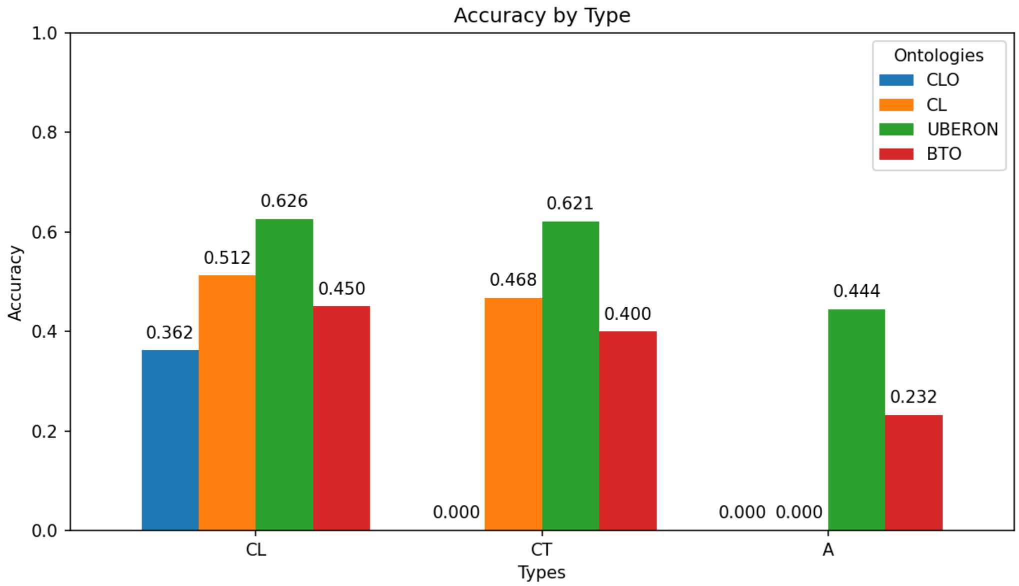

Supplementary Figure 3: Accuracy of the fine-tuned model for each ontology according to the type of concept associated with the label.
